# Magnesium Borohydride: From Hydrogen Storage to Magnesium Battery**

**DOI:** 10.1002/anie.201204913

**Published:** 2012-08-21

**Authors:** Rana Mohtadi, Masaki Matsui, Timothy S Arthur, Son-Jong Hwang

**Affiliations:** Dr. R. Mohtadi, Dr. M. Matsui, Dr. T. S. Arthur Materials Research Department Toyota Research Institute of North AmericaAnn Arbor, MI 48105 (USA); Division of Chemistry and Chemical Engineering California Institute of Technology(USA)

**Keywords:** borohydrides, electrochemistry, hydrogen, magnesium

Since Bogdanović and Schwickardi illustrated the possibility of reversibly storing hydrogen in sodium alanate,^[1]^ extensive research efforts have been dedicated to investigating the hydrogen storage potential of complex metal hydrides. In particular, borohydrides have attracted great interest because of their superior gravimetric hydrogen content.^[2]^ Of these, magnesium borohydride Mg(BH_4_)_2_, first reported in 1950^[3]^ and more recently studied for hydrogen storage, has attracted attention because of its relatively low hydrogen-release temperature and reversibility.^[2a]^, ^[4]^ Furthermore, borohydrides are strong reducing agents that are widely used in organic and inorganic syntheses. This reducing power translates to high stability against electrochemical reduction; this stability could be exploited in highly reductive environments, such as battery anodes. Therefore, for the first time, we have conducted research towards harnessing this property of borohydrides for their use in rechargeable batteries. In particular, we have been focusing on utilizing a Mg(BH_4_)_2_ based electrolyte in a rechargeable magnesium battery.

Recently, magnesium batteries have received increased attention as alternatives to the lithium-based battery because of the high volumetric capacity (3832 mA h cm^−3^), improved safety (nondendritic), and abundance of Mg metal.^[5]^ Despite the potential of Mg batteries, several key challenges need to be overcome for this technology to become viable. For instance, current state-of-the-art electrolytes use organomagnesium salts and complexes as they are the only ones known to be compatible with the Mg anode that allow for reversible electrochemical Mg deposition and stripping.^[5b]^, ^[6]^ Although some of these electrolytes have shown impressive stability against electrochemical oxidation, they were also found to be corrosive.^[6]^ This property was attributed to the presence of chlorides in either/both their cations and anions.^[6]^ Conventional inorganic and ionic salts such as Mg(ClO_4_)_2_ were found to be incompatible with the Mg anode as a result of the formation of an ion-blocking layer formed by their electrochemical reduction.^[6]^ Hence, the discovery of halide-free electrolytes with high reductive stabilities is crucial for realizing a practical rechargeable Mg battery system.

Herein, we propose a new class of electrolytes based on Mg(BH_4_)_2_ for a Mg battery. We show the first example of electrochemical reversible Mg deposition/stripping in a halide-free inorganic salt in both tetrahydrofuran (THF) and dimethoxyethane (DME) solvents. An increase of several orders of magnitude in the current densities, and high coulombic efficiencies of up to 94 % are observed in DME when LiBH_4_ is used as an additive. Furthermore, we use this electrolyte in a rechargeable Mg battery, thus giving the first example of a borohydride electrolyte in a battery system. This work also illustrates the unique properties of borohydrides and opens the door for designing a whole new class of electrolytes for Mg batteries.

Mg deposition/stripping was studied for Mg(BH_4_)_2_ in ether solvents. Figure 1 a shows the cyclic voltammogram obtained for 0.5 M Mg(BH_4_)_2_/THF where a reversible reduction–oxidation process took place with onsets at −0.6 V/0.2 V and a 40 % coulombic efficiency (Figure 1 a, inset), thus indicating reversible Mg deposition and stripping. X-ray diffraction (XRD) confirmed that the deposited product from the galvanostatic reduction of the above solution (Figure 1 b) was hexagonal Mg, hereby establishing the compatibility of Mg(BH_4_)_2_ with Mg metal. The electrochemical oxidative stabilities measured on platinum, stainless steel, and glassy carbon electrodes were 1.7, 2.2, and 2.3 V, respectively (Figure S7). These results showed that for the first time: 1) Mg(BH_4_)_2_ is electrochemically active in THF, that is, ionic conduction is possible, and 2) reversible magnesium deposition/stripping from an inorganic, relatively ionic (Mg Bader charge is +1.67)^[7]^ and halide-free salt is feasible. Although these results are promising, to make this electrolyte more practical for use in batteries the electrochemical performance needs to be improved by lowering the overpotentials, and achieving higher current density and coulombic efficiency. In addition, the demonstration of this performance in less-volatile solvents would make Mg(BH_4_)_2_ based electrolytes even more practical. Therefore, DME was selected (its boiling temperature is 19 °C higher than that of THF) for further investigations. The cyclic voltammogram obtained for 0.1 M Mg(BH_4_)_2_/DME is shown in Figure 1 c where a substantial improvement in the electrochemical performance compared to Mg(BH_4_)_2_/THF was evident from: 1) a 10-fold increase in the current density, 2) a reduction in the overpotentials (deposition/stripping onsets at −0.34 V/0.03 V versus −0.6 V/0.2 V in THF), and 3) a higher coulombic efficiency of 67 % (40 % in THF). These findings suggested that the Mg electroactive species was present in higher concentration and had increased mobility in DME despite the lower solubility of Mg(BH_4_)_2_ in DME versus THF.

**Figure 1:**
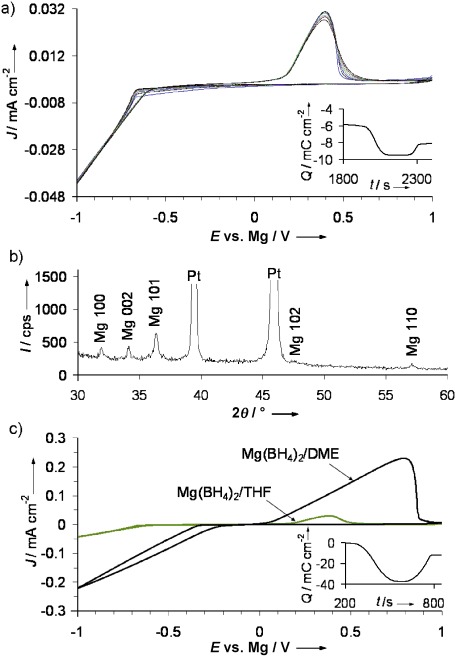
For 0.5 M Mg(BH_4_)_2_/THF: a) Cyclic voltammogram (8 cycles), inset shows deposition/stripping charge balance (third cycle), and b) XRD results following galvanostatic deposition of Mg on a Pt working electrode. c) Cyclic voltammogram for 0.1 M Mg(BH_4_)_2_/DME compared to 0.5 M Mg(BH_4_)_2_/THF. Inset shows deposition/stripping charge balance for Mg(BH_4_)_2_/DME. All experiments used Pt working electrode and Mg reference/counter electrodes.

These results demonstrated that for the Mg(BH_4_)_2_ electrolyte, the electrochemical performance in DME is higher than that in THF. In contrast, organomagnesium electrolytes show an optimum electrochemical performance in THF.5b To further improve the electrochemical performance, it was pertinent to characterize the electroactive species in Mg(BH_4_)_2_ solutions. Therefore, IR and NMR spectroscopic analyses (Figure 2) were conducted for 0.5 M Mg(BH_4_)_2_/THF and 0.1 M Mg(BH_4_)_2_/DME. The IR B–H stretching region (2000–2500 cm^−1^) showed two strong widely separated bands (Mg(BH_4_)_2_/THF: 2379 cm^−1^, 2176 cm^−1^ and Mg(BH_4_)_2_/DME: 2372 cm^−1^, 2175 cm^−1^); note that the spectra for 0.1 and 0.5 M of Mg(BH_4_)_2_ in THF are similar (Figure S2). These IR spectra are similar to those of covalent borohydrides^[8]^ and those of Mg(BH_4_)_2_ solvates from THF and diethyl ether^[9]^ where two hydrogen atoms in BH_4_^−^ are forming a bridge to one metal atom (μ bonding). Therefore, we assigned the bands at the higher and lower B–H frequencies to terminal and bridging B–H vibrations (B–H_t_ and B–H_b_), respectively. The band and shoulder at 2304 and 2240 cm^−1^ were assigned to asymmetric B–H_t_ and B–H_b_ vibrations, respectively. As complete dissociation of Mg(BH_4_)_2_ into discreet ions is unlikely (as other borohydrides are in ethers),^[10]^ we propose that Mg(BH_4_)_2_ is present as the contact ion pair Mg[(μ-H)_2_BH_2_]_2_, which partially dissociates into [Mg{(μ-H)_2_BH_2_}]^+^ and BH_4_^−^ as in [Eq. (1)]; since the different B–H bands most likely overlap, it is not possible to distinguish all the species.

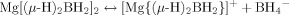
1

**Figure 2:**
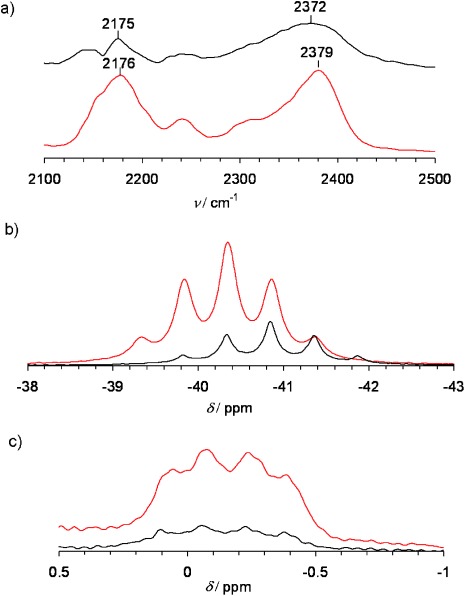
For Mg(BH_4_)_2_ in THF (red line) and in DME (black line): a) IR spectra, b) ^11^B NMR spectra, and c) ^1^H NMR spectra.

Where [Mg{(μ-H)_2_BH_2_}]^+^ may further dissociate:

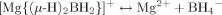
2

For the spectrum of Mg(BH_4_)_2_/DME, although the main features present in the spectrum of Mg(BH_4_)_2_/THF were retained, the νB–H_t_ band is broader and shifted to a lower value and the νB–H_b_ intensity is relatively weaker. Although νB–H_t_ band broadening suggests a pronounced presence of a species similar to that found in THF, the shift in the band maximum indicates a more-ionic B–H bond (the νB–H_t_ shift is similar to those resulting from BH_4_^−^ ions that have enhanced ionic character, such as in stabilized covalent borohydrides).^[8]^ In addition, the relative weakening in νB–H_b_ intensity suggests that there is more free BH_4_^−^. The NMR spectrum of BH_4_^−^ in DME (Figure 2 b and c) indicates that there is increased boron shielding as the associated signal is shifted by about 0.5 ppm (quintet in ^11^B NMR spectrum), and slightly reduced proton shielding (0.01 ppm, quartet in ^1^H NMR spectrum); these results are consistent with B–H bonds that have a higher ionic character than those in BH_4_^−^ in THF (distinguishing B–H_t_ from B–H_b_ is not possible likely because of rapid hydrogen exchange). These findings are evidence of weaker interactions between Mg^2+^ and BH_4_^−^ within the ion pair and an enhanced dissociation in DME [Eq. (1) and (2)]. So despite the fact that DME has a slightly lower dielectric constant (7.2) compared to THF (7.4), its chelation properties (owing to the presence of two oxygen sites per molecule)^[11]^ resulted in an enhanced dissociation and thus an improved electrochemical performance.

Based on the understanding gained of the nature of Mg(BH_4_)_2_ in solution, we hypothesized that electrochemical performance would be enhanced when the association within the ion pair is weakened. To achieve this, an additive that has an acidic cation with the following characteristics is desirable: 1) reductive stability comparable to Mg(BH_4_)_2_, 2) nonreactive, 3) halide free, and 4) soluble in DME. Hence, LiBH_4_ was selected as it fulfils all of the above criteria. Mg deposition and stripping was studied in DME using different molar ratios of LiBH_4_ to Mg(BH_4_)_2_; Figure 3 a shows the cyclic voltammogram obtained for 3.3:1 molar LiBH_4_ to Mg(BH_4_)_2_ (Figure S8a and S8b show the cyclic voltammograms for different concentrations). The use of LiBH_4_ resulted in an increase of two orders of magnitude in the current density (i.e. oxidation peak current Jp=26 mA cm^−2^), and in a higher coulombic efficiency of up to 94 %. We attribute the deposition/stripping currents solely to Mg because of the absence of Li after galvanostatic deposition (Figure 3 b), and also the lack of electrochemical activity in a LiBH_4_/DME solution (Figure S8a). The ionic character of BH_4_^−^ was enhanced, as evidenced by lower νB–H_t_ and higher νB–H_b_ bands in the IR spectrum (Figure 3 c), thus implying that LiBH_4_ has a role in increasing Mg(BH_4_)_2_ dissociation (the B–H bands for LiBH_4_/DME occur at lower values, Figure S9). The coulombic efficiency was proportional to the molar ratios of LiBH_4_/Mg(BH_4_)_2_ (Figure S10). A rechargeable Mg battery with a Chevrel phase Mo_6_S_8_ cathode, an Mg metal anode, and this optimized electrolyte (Figure 4) demonstrated reversible cycling capabilities at a 128.8 mA g^−1^ rate (capacity retention and cathode magnesiation are shown in Figure S11 and Figure S12). We are currently investigating the sources of the overcharge and capacity fade.

**Figure 3:**
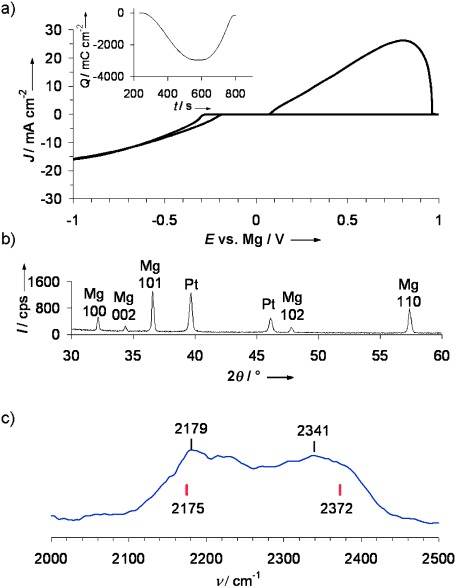
For LiBH_4_ (0.6 M)/Mg(BH_4_)_2_ (0.18 M) in DME: a) Cyclic voltammogram (inset shows deposition/stripping charge balance). b) XRD results following galvanostatic deposition of Mg on a Pt disk. c) IR spectra (red | indicates band maxima for Mg(BH_4_)_2_/DME).

**Figure 4:**
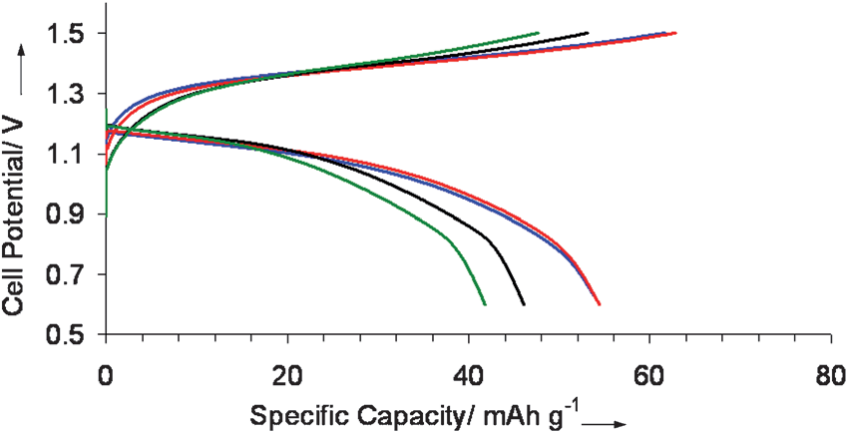
Charge/discharge profiles with Mg anode/Chevrel phase cathode for 3.3 molar LiBH_4_/Mg(BH_4_)_2_ in DME. Cycle 1 (blue), cycle 2 (red), cycle 20 (black), cycle 40 (green).

In summary, unprecedented reversible Mg deposition and stripping from an inorganic and relatively ionic salt was obtained in THF and DME. Higher current density and lower overpotentials were achieved in DME compared to those in THF. Substantial enhancement in the coulombic efficiency and the current density was accomplished by the addition of LiBH_4_. Battery performance was demonstrated using a Chevrel phase cathode. Although the oxidative stability (1.7 V vs. Mg on platinum) currently limits Mg(BH_4_)_2_ utilization with high voltage cathodes, reversibility in the absence of halides and THF makes this salt extremely unique and these findings very important for designing a whole new class of Mg(BH_4_)_2_ based electrolytes. Currently, we are investigating improving the oxidative stability of Mg(BH_4_)_2_. In addition, the exact nature of the electroactive species in the presence and the absence of the additive is being studied to guide the design of Mg(BH_4_)_2_ based electrolytes. This work provides a stepping stone for extending the applications of Mg(BH_4_)_2_ and underscores the beauty and versatility of the chemistry of borohydrides.

## Experimental Section

Magnesium borohydride (Mg(BH_4_)_2_, 95 %) lithium borohydride (LiBH_4_, 90 %), anhydrous tetrahydrofuran (THF), and dimethoxyethane (DME) were purchased from Sigma–Aldrich. Cyclic voltammetry was conducted in a three-electrode cell with Mg wire/ribbon as reference/counter electrodes. The electrochemical testing was conducted in an argon filled glovebox with O_2_ and H_2_O amounts kept below 0.1 ppm. Details of the analyses and battery testing conducted are described in the Supporting Information.

## Notes

Detailed facts of importance to specialist readers are published as ”Supporting Information”. Such documents are peer-reviewed, but not copy-edited or typeset. They are made available as submitted by the authors.
