# Supplementary material for: Magnesium Borohydride: From Hydrogen Storage to Magnesium Battery
Source: Angew Chem Int Ed Engl. 2012 Aug 21;51(39):9780–3. doi: 10.1002/anie.201204913 (PMC3588139; doi:10.1002/anie.201204913)
Supplement: Supplementary file 1 [file anie0051-9780-sd1.pdf]

Angewandte Chemie
Eine Zeitschrift der Gesellschaft Deutscher Chemiker

Supporting Information
© Wiley-VCH 2012
69451 Weinheim, Germany

Magnesium Borohydride: From Hydrogen Storage to Magnesium Battery**

Rana Mohtadi, Masaki Matsui, Timothy S. Arthur, and Son-Jong Hwang*

anie_201204913_sm_miscellaneous_information.pdf

Table of Contents

Experimental details for sample preparation and instrumental analysis **P.2-4**

Infrared spectra of solid magnesium borohydride and lithium borohydride **(Figure S1)** **P.5**

Infrared spectra for magnesium borohydride in THF **(Figure S2)** **P.5**

Infrared spectra for magnesium borohydride in DME **(Figure S3)** **P.6**

Nuclear Magnetic Resonance of ^{11}B for magnesium borohydride in DME and THF **(Figure S4)** **P.6**

Nuclear Magnetic Resonance of ^{1}H for magnesium borohydride in DME and THF **(Figure S5)** **P.7**

Infrared spectra comparing magnesium borohydride and lithium borohydride in DME **(Figure S6)** **P.7**

Electrochemical oxidative stability of $Mg(BH_4)_2$/THF measured on a platinum, 316 stainless steel and glassy carbon electrodes **(Figure S7)** **P.8**

Cyclic voltammetry comparing Mg deposition/stripping for electrolytes at different magnesium borohydride and lithium borohydride concentrations in DME **(Figure S8)** **P.8**

Comparison of the IR spectra obtained for lithium borohydride/DME, magnesium borohydride/DME and lithium borohydride/magnesium borohydride /DME **(Figure S9)** **P.9**

Effect of lithium borohydride concentration on the coulombic efficiency **(Figure S10)** **P.9**

Discharge and charge capacity retention for a battery cell **(Figure S11)** **P.10**

X-ray diffraction obtained for the cathode prior to and after the 1st battery discharge demonstrating the magnesiation of Chevrel phase Mo_6S_8 **(Figure S12)** **P.10**

References **P.11**

Experimental details for sample preparation and instrumental analysis

All sample handling was conducted in mbraun glove box with O_2 and H_2O content kept to less than 0.1 ppm. Magnesium borohydride (95%), lithium borohydride(90%), anhydrous tetrahydrofuran (THF) and anhydrous dimethoxyethane (DME) were all purchased from SigmAldrich and all used as received. Samples received were analyzed using infrared and elemental analysis prior to usage. In all experiments, a predetermined amount of magnesium borohydride was weighed and allowed to stir in the solvent at room temperature for at least 2 hours. All excess amounts were removed by filtering the solution prepared so that a clear solution was used in all further experimentation. Mass balance showed that the concentration of magnesium borohydride was as follows: 0.5 M in THF, 0.1 M in DME and 0.18 M in solution of DME with $LiBH_4$.

Elemental Analysis
As received samples were analyzed for impurities presence and elemental analysis was also conducted following sample dissolution and filtration. The elemental analysis was conducted at Galbraith Laboratories Inc. The following results were obtained:

$Mg(BH_4)_2$ solid: Cl^{-1}: 0.25%, F^{-1}: <419 ppm, C: 1.54%, Li^+: <193 ppm
$LiBH_4$ solid: Cl^{-1}<52 ppm, F^{-1}: <0.16%, C: <0.5%, Mg^{+2}: <0.21%
0.5 M $Mg(BH_4)_2$/THF: Cl^{-1}:158 ppm, F^{-1}: <167 ppm, Mg^{+2}: 1.12%
0.1 M $Mg(BH_4)_2$/DME: Cl^{-1} <49 ppm, F^{-1}<14 ppm, Mg^{+2}: 0.658%
0.25 M $LiBH_4$/DME: Cl^{-1}:54 ppm, F^{-1}<30 ppm, Li^+: 0.24%

Electrochemical testing
The electrochemical testing was conducted in a three electrodes BASi 4 drams shell vial placed inside mbraun glove box at 25 ˚C at less than 0.1 ppm O_2 and H_2O content. The working electrodes used included platinum (0.02 cm^2), glassy carbon (.077 cm^2) and 316 stainless steel (.0341 cm^2). All the electrodes were polished, sonicated and kept in a dry vacuum oven prior to each experiment. A magnesium wire and ribbon (BASi) were used as reference and counter electrodes, respectively. Oxides which may form on the surface of Mg electrodes during storage were removed prior to each experiment by rubbing their surface with a glass slide. Electrochemical testing was conducted using a BioLogic potentiostat ran at a scan rate of 5 mV s^{-1}. Galvanostatic deposition of magnesium was conducted on a 1.5 cm diameter, 0.01 cm thick polished platinum disk (99.997% Permion® Alpha Aesar) held at current corresponding to -0.7 V vs. magnesium. Figure ES1 shows typical raw data directly measured for the oxidation and reduction currents function of time during cycling voltammetry voltage scan and the charge balance calculated using the EC-lab Software®. The coulombic efficiency was calculated by dividing the oxidation charge over the reduction charge. As an example, the coulombic efficiency for 0.1 M $Mg(BH_4)_2$/DME (Figure ES1) was calculated as follows: charge consumed in reduction=-.745 mC, charge produced from oxidation=.502 mC, coulombic efficiency= $|.500/-.745|$*100%=67.3%. Since the reference electrode used was a magnesium wire, the electrochemical testing was conducted in the voltage range between -1 and 1 V in order to limit the electrochemical activity solely to magnesium deposition and stripping.

a.

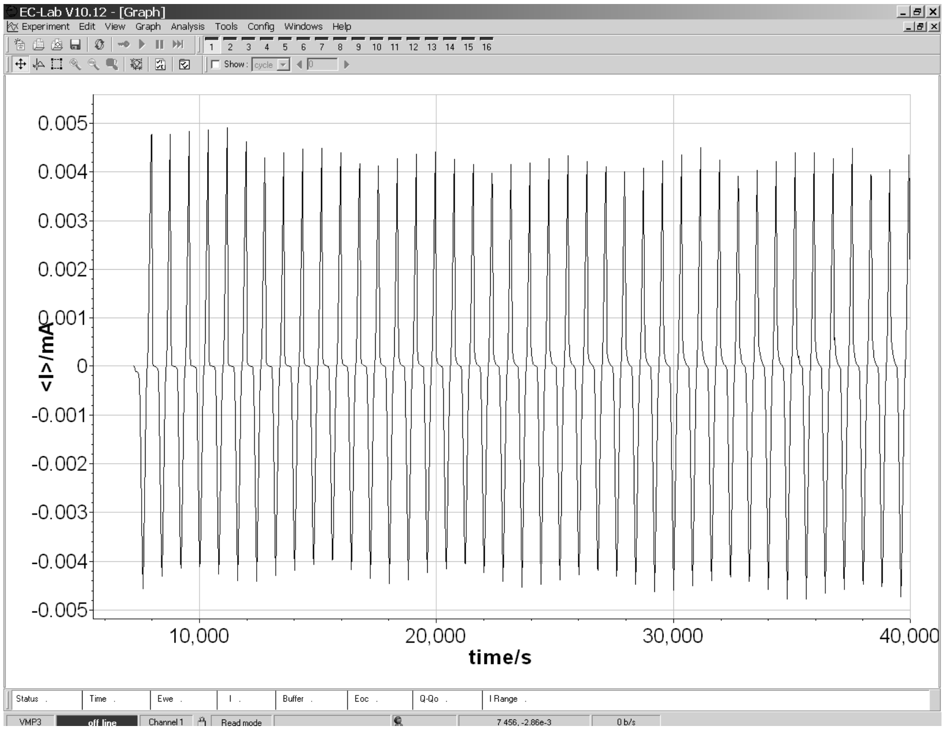

b.

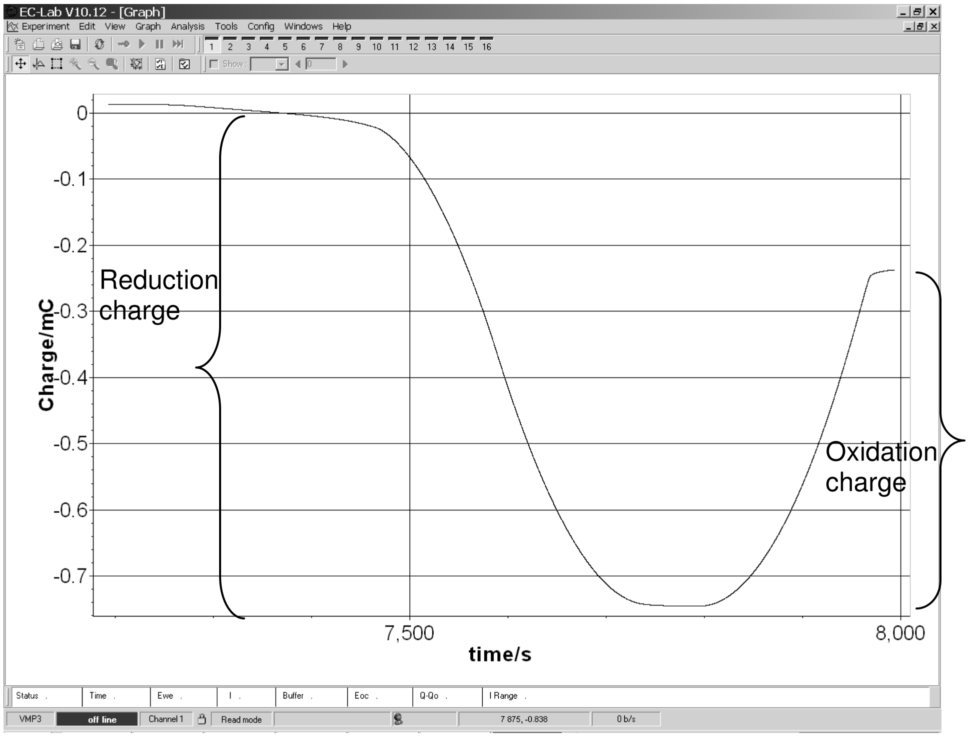

Figure ES1: Raw data obtained during cyclic voltammetry experiment conducted for 0.1 M magnesium borohydride/DME depicting a) the current measured with time during voltage cycling and b) the reduction/oxidation charge balance calculated for the 1st cycle.

The onset for the oxidation voltage was determined by the turning point at which a rapid increase in the current density was observed.
The battery testing was conducted in a two electrode TomCell (TomCell Japan) with a Mg metal anode and Mo_6S_8 cathode (prepared according to the procedure presented in reference 1). A glass fiber separator (Whatman® .25 mm filterpaper) and 200 µl of electrolyte was used. The cathode consisted of 70% Mo_6S_8, 20% carbon black and 10% PTFE binder. The cell was assembled in the glove box and ran at room temperature.

Xray Diffraction
Xray diffraction was conducted in Rigaku SmartLab® equipped with Cu Kα Xray source. For all Xray analysis, the platinum disk obtained following galvanostatic deposition of magnesium was carefully washed with the respective solvent, dried then covered with 8 µm Kapton film to prevent exposure to air. All experiments were ran at 0.3˚ /min scan rate and 0.02˚ step size.

Infrared analysis
To prevent exposure to air during the analysis, an air tight Specac Smart Golden Gate ATR™ cell equipped with a diamond crystal was used in all IR analysis which was run using a Nicolet 8700 FTIR (by Thermo Scientific).

Nuclear Magnetic Resonance NMR
Nuclear magnetic resonance (NMR) measurements were performed using a Bruker Avance 500MHz spectrometer (11.7 T) equipped with a boron-free Bruker 4mm CPMAS probe. About 90 micro-litters of solution samples were loaded into a 4mm ZrO_2 rotor and sealed with a tightly fitting kel-F cap inside an argon glove box, and inserted into NMR probe under N_2 flowing condition. Single pulse spectra were acquired at static condition for ^1H and ^{11}B, of which operating frequencies are 500.2 and 160.5 MHz, respectively. A 4 micro-sec 90 degree pulse was applied for both nuclei and ^1H decoupling was not applied during ^{11}B measurements in order to avoid the radio frequency heating. The spectra were reported in part per million (ppm) with referenced to external standards of tetramethylsilane (TMS) and $BF_3{\cdot}O(CH_2CH_3)_2$ for ^1H and ^{11}B, respectively.

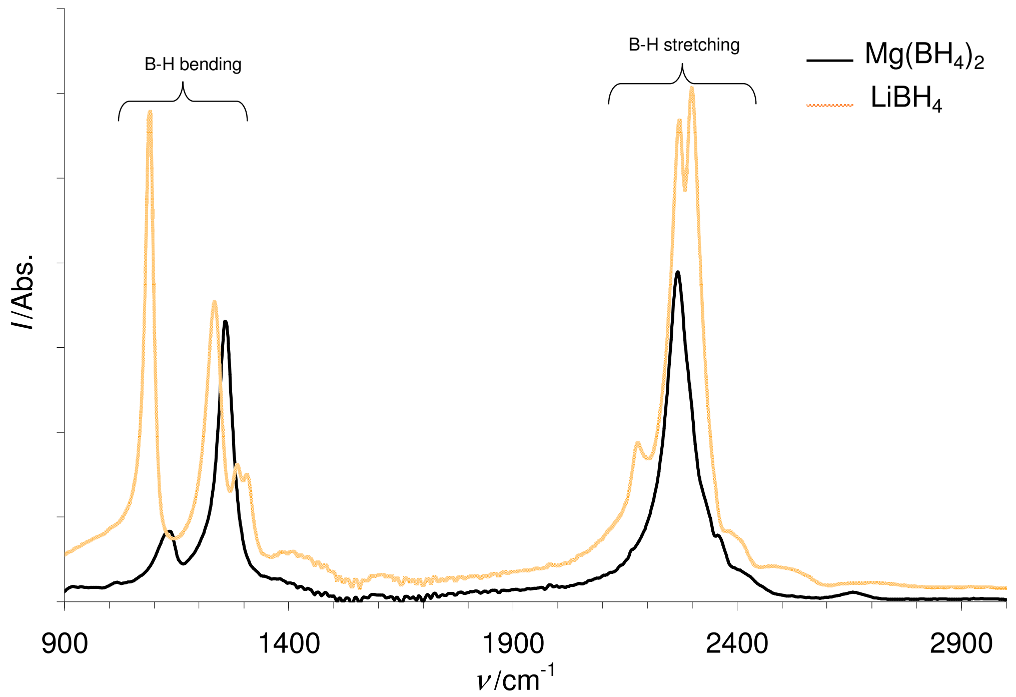

Figure S1: Infrared spectra of solid magnesium and lithium borohydride. The bands between 2000-2500 cm^{-1} represents B-H stretching vibrations and between 1000-1400 cm^{-1} represents bending B-H modes [2,3]

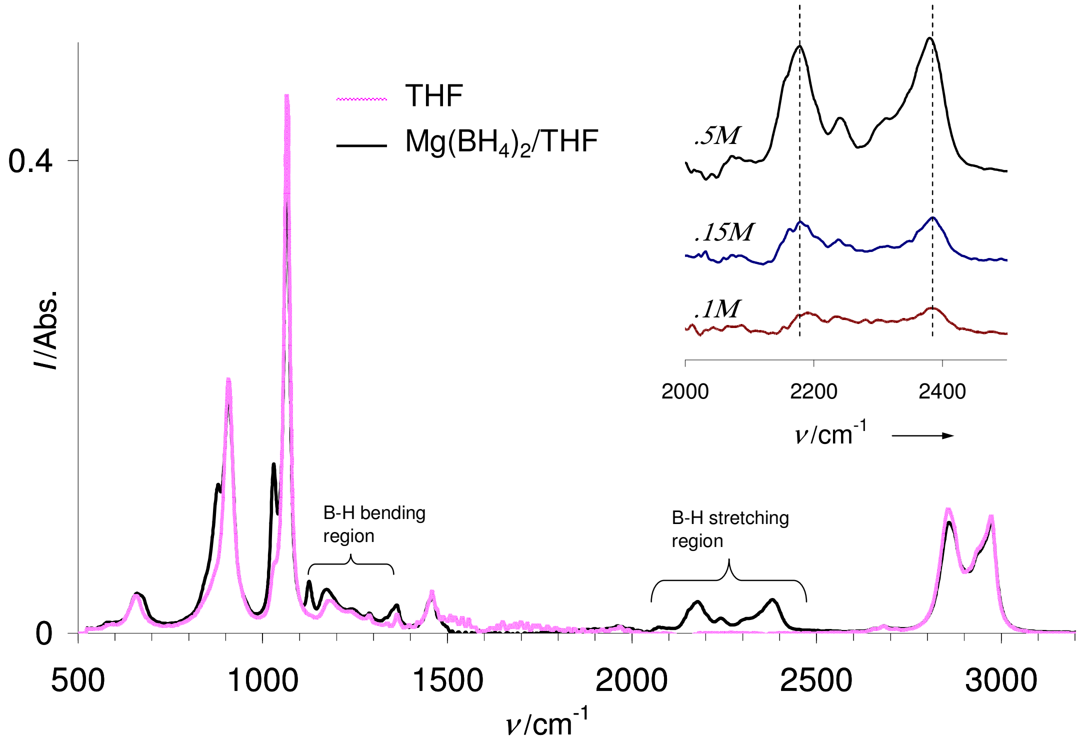

Figure S2: Infrared spectra for 0.5 M magnesium borohydride in THF. THF spectra is shown for reference. The inset compares the B-H stretching region for 0.5, 0.15 and 0.1 M $Mg(BH_4)_2$/THF solutions to demonstrate the absence of discernable changes in the relative peak ratios of $\nu B\text{-}H_t$ to $\nu B\text{-}H_b$ and in the bands positions.

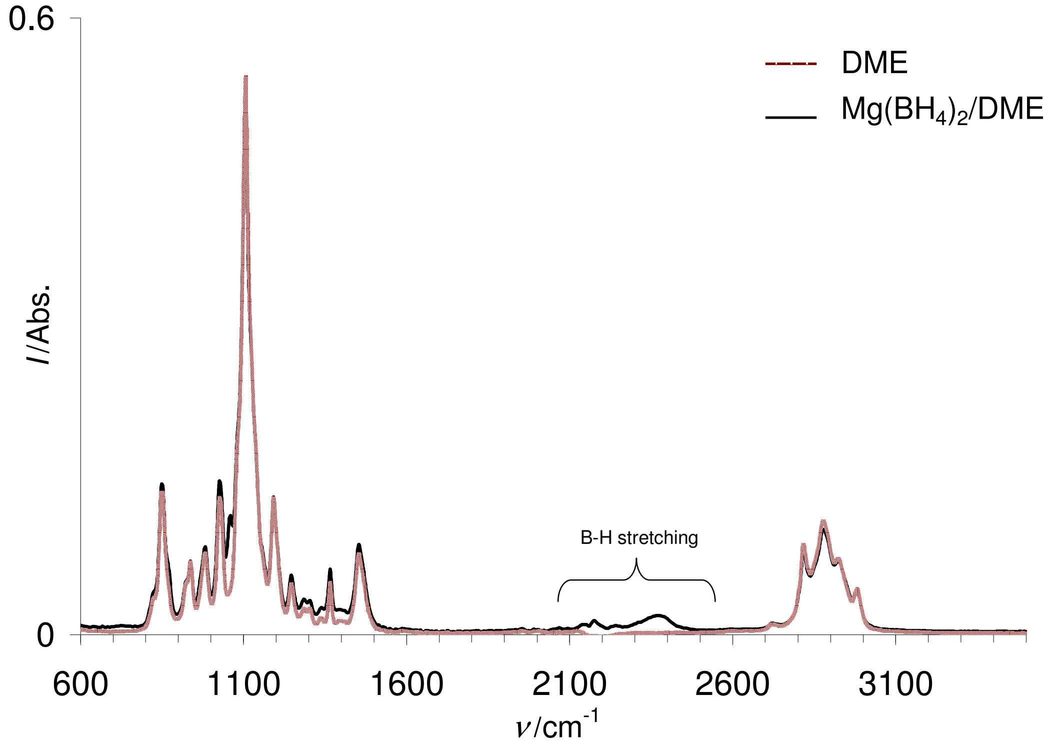

Figure S3: Infrared spectra for 0.1 M magnesium borohydride/DME. DME spectra is shown for reference.

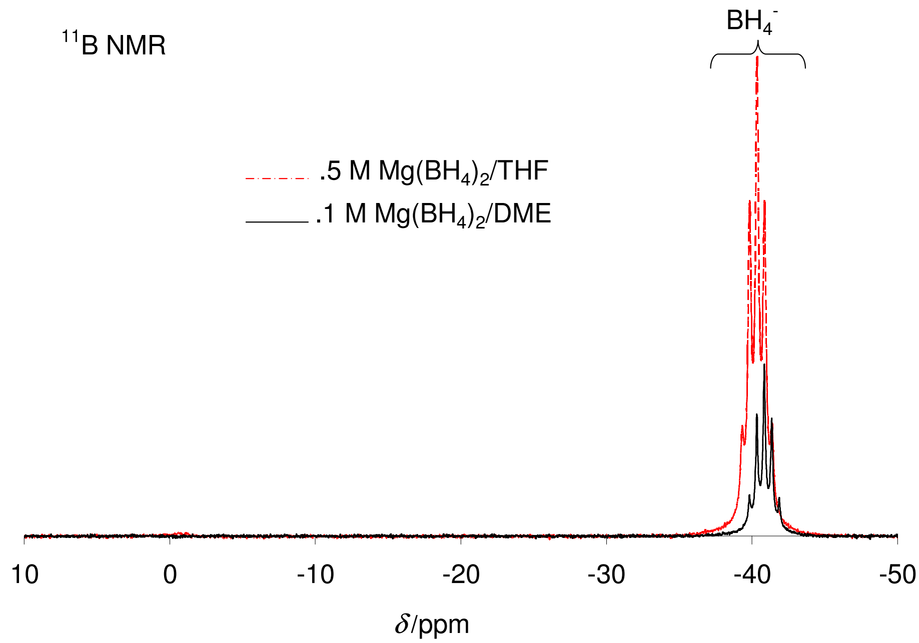

Figure S4: ^{11}B NMR (full range) for 0.1 M magnesium borohydride/DME and 0.5 M magnesium borohydride/THF.

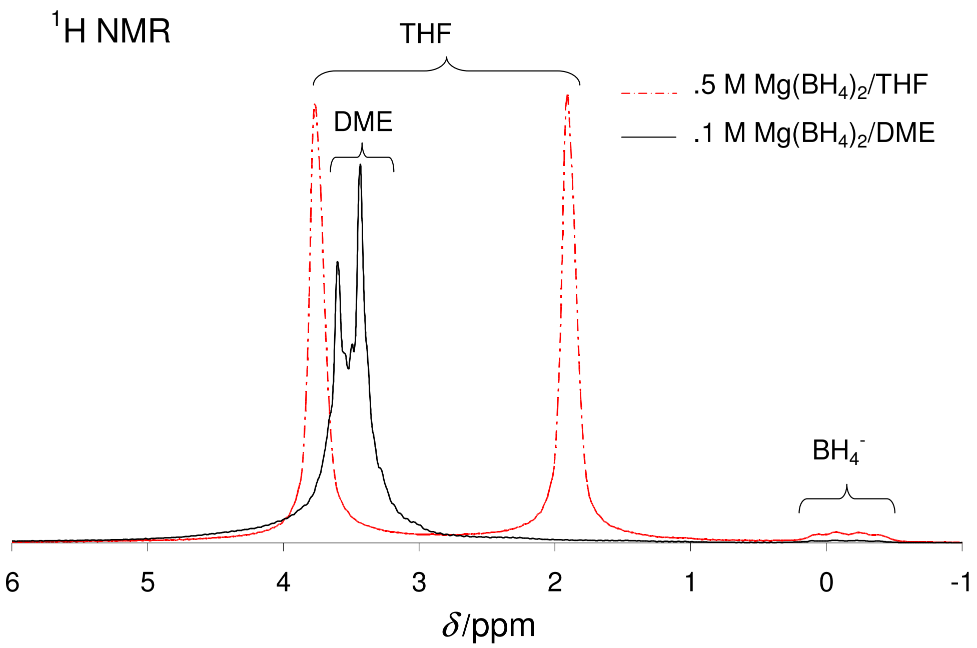

Figure S5: ^{1}H NMR (full range) for 0.1 M magnesium borohydride/DME and 0.5 M magnesium borohydride/THF. J(B-H) ~ 80 Hz.

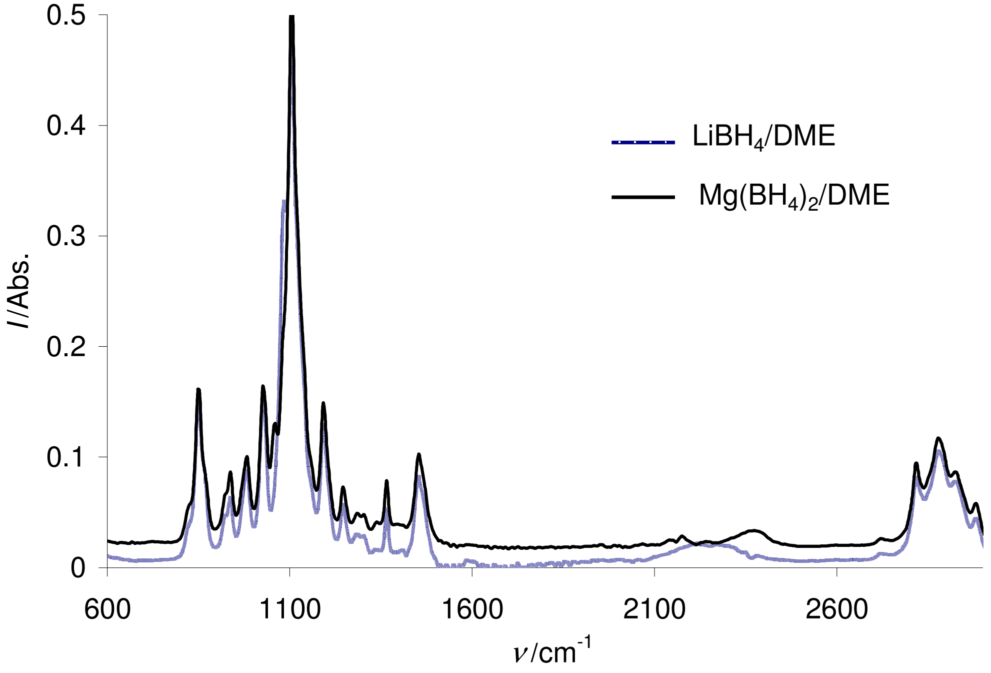

Figure S6: Infrared spectra comparing .1 M magnesium borohydride/DME to 0.6 M lithium borohydride/DME.

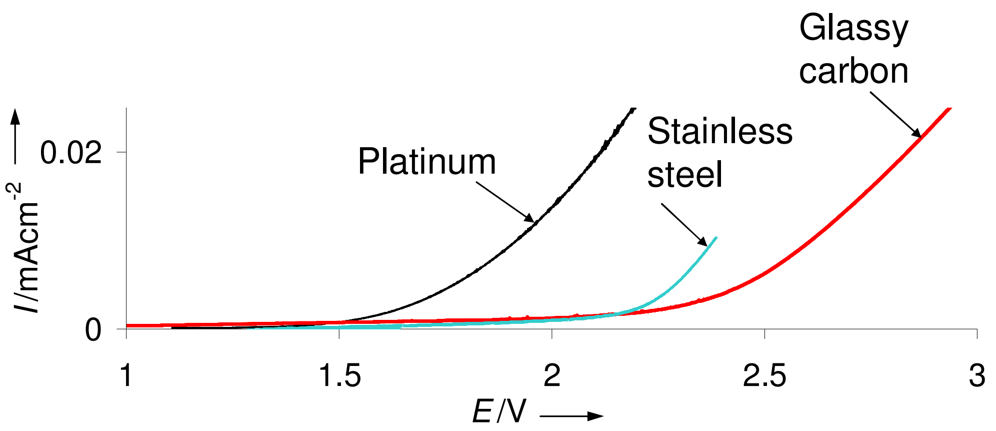

Figure S7: Electrochemical oxidative stability of $Mg(BH_4)_2$/THF measured on a platinum, 316 stainless steel and glassy carbon electrodes. Similar stabilities were obtained for $Mg(BH_4)_2$ electrolytes in DME.

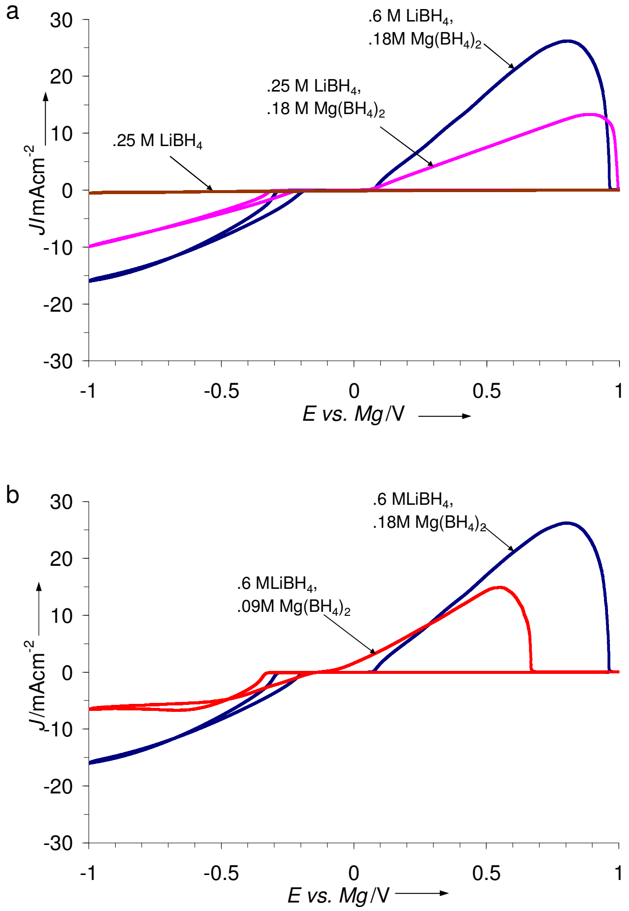

Figure S8: a) Effect of $LiBH_4$ on the electrochemical performance of $Mg(BH_4)_2$/DME at .25 M and .6 M concentrations (1.4 and 3.3 molar ratio to $Mg(BH_4)_2$). The cyclic voltammetry for 0.25 M $LiBH_4$/DME is shown for reference to illustrate the absence of any electrochemical activity, b) Demonstration of $LiBH_4$ (0.6M) effects in .09 and .18 M $Mg(BH_4)_2$ /DME showing an improved electrochemical performance at higher $Mg(BH_4)_2$ concentration.

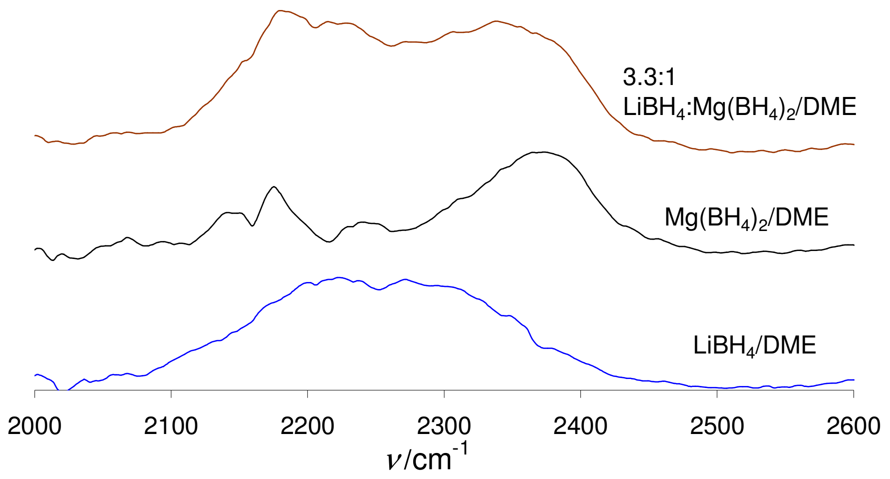

Figure S9: Comparison of the IR spectra obtained for 0.6 M $LiBH_4$/DME, .1 M $Mg(BH_4)_2$/DME and 0.6 M $LiBH_4$/.18 M $Mg(BH_4)_2$/DME. Enhanced BH_4^- ionicity in 0.18 M $Mg(BH_4)_2$/0.6 M $LiBH_4$/DME electrolyte vs. 0.1 M $Mg(BH_4)_2$/DME found. Note that the B-H band in $LiBH_4$/DME exhibits a broad large peak indicating the presence of several species[4] (i.e. dissociation) which seems to be of higher ionic character compared to the B-H in $Mg(BH_4)_2$/DME and $Mg(BH_4)_2$/$LiBH_4$/DME.

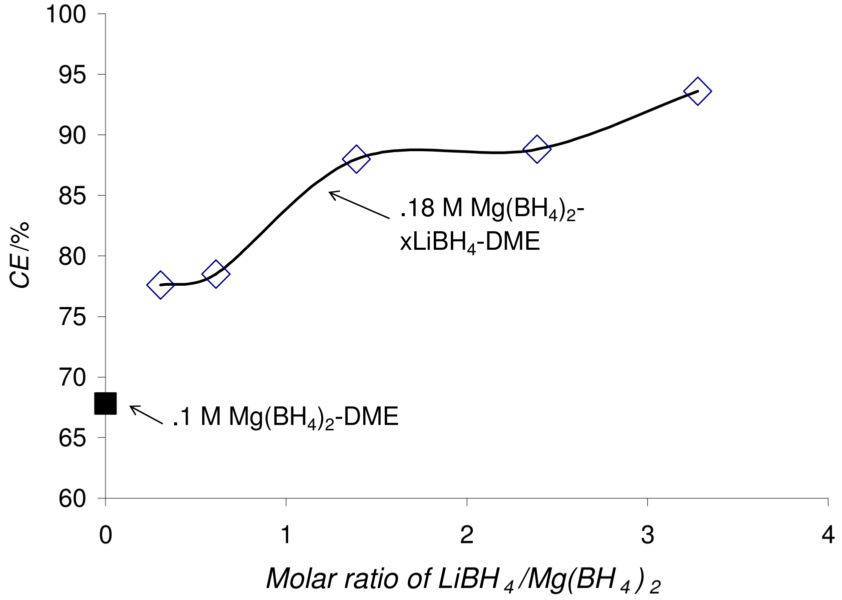

Figure S10: Effect of lithium borohydride concentration on the coulombic efficiency. A proportional relation exits between $LiBH_4$ amount the coulombic efficiency.

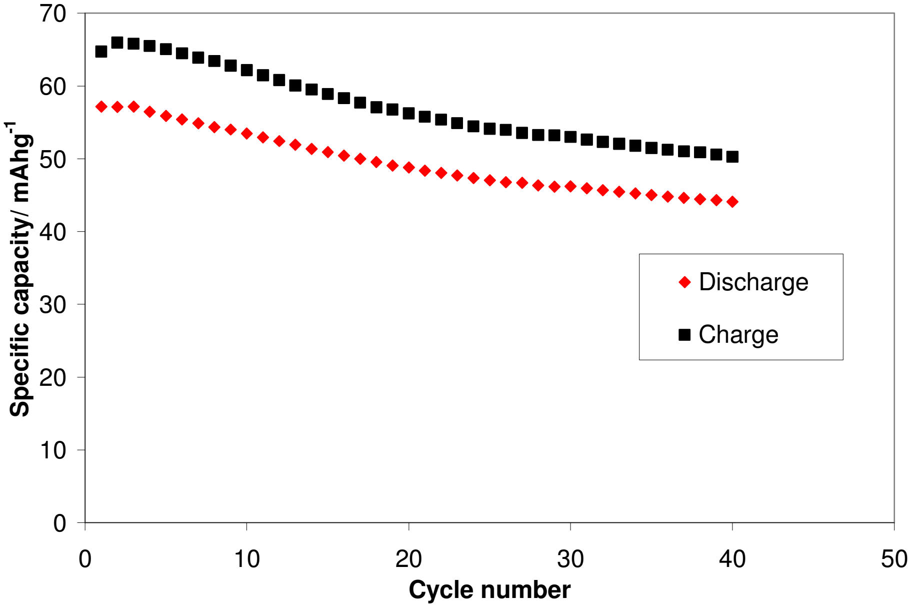

Figure S11: Discharge and charge capacity retention (40 cycles) for a battery cell consisting of Mg metal anode, Mo_6S_8 cathode and 0.18 M $Mg(BH_4)_2$/0.6 M $LiBH_4$/DME electrolyte as function of cycle number. The cell was operated at a current rate of 128.8 mAg^{-1} at room temperature. (Mo_6S_8 theoretical capacity is 122 mAh g^{-1} and in order to experimentally get a reversible specific capacity closer to the theoretical value, optimization of the cathode preparation, lowering the charging rates and increasing the charging voltages would be needed).

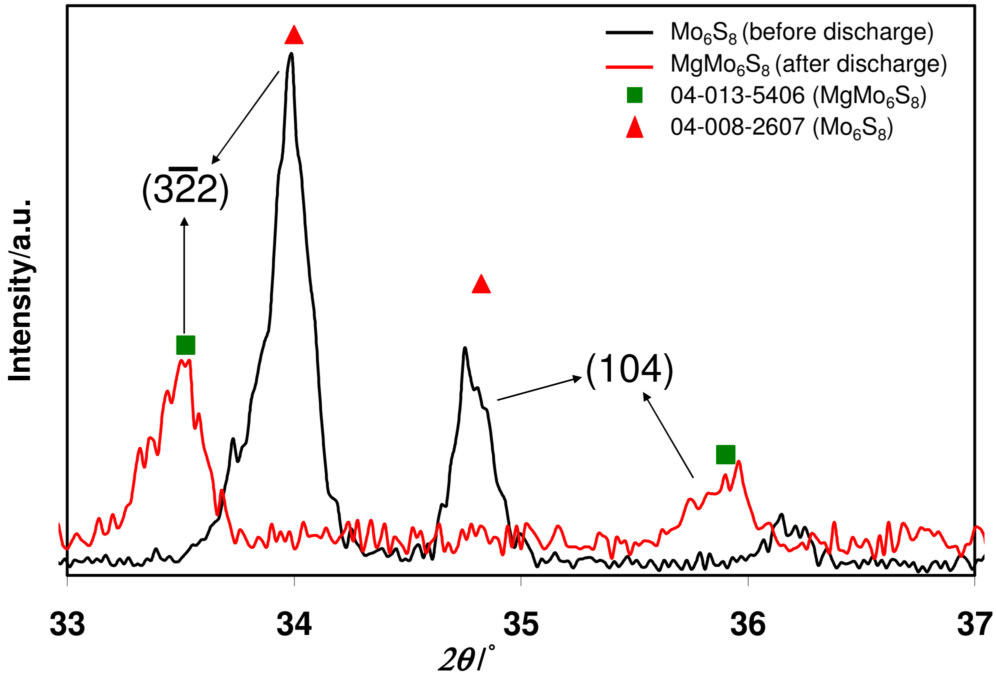

Figure S12: X-ray diffraction obtained for the Chevrel phase cathode prior to (—) and after the 1st battery discharge (—) demonstrating the magnesiation of the Chevrel phase Mo_6S_8[5].

Reference

[1] E. Lancry, E. Levi, A. Mitelman, S. Malovany, D. Aurbach, Molten salt synthesis (MSS) of $Cu_2Mo_6S_8$—New way for large-scale production of Chevrel phases, Journal of Solid State Chem. 2006, 179, 1879–1882.

[2] W.C. Price, The Infra-Red Absorption Spectra of Some Metal Borohydrides, Journal of Chemical Physics 1949, 17(11), 1044-1053.

[3] a) Y. Filinchuk, R. Černý, H. Hagemann, Insight into $Mg(BH_4)_2$ with Synchrotron X-ray Diffraction: Structure Revision, Crystal Chemistry, and Anomalous Thermal Expansion, Chem. Mater., 2009, 21(5), 925–933; b) Y. Filinchuk, Bo Richter, T. R. Jensen, V. Dmitriev, D. Chernyshov, and H. Hagemann, Porous and Dense Magnesium Borohydride Frameworks: Synthesis, Stability, and Reversible Absorption of Guest Species, Angew. Chem. Int. Ed. 2011, 50, 11162 –11166.

[4] I. M. Strauss, M.C.R. Symons, V.K. Thompson, Solvation Spectra Part 53. -Infrared and Nuclear Magnetic Resonance Studies of the Tetrahydroborate Anion in various Pure Solvents and Binary Aqueous Mixtures, J. Chem. Soc., Faraday Trans.1 1977,73, 1253-1259.

[5] E. Lancry, E. Levi, Y. Gofer, M. Levi, G. Salitra, D. Aurbach, Leaching Chemistry and the Performance of the Mo_6S_8 Cathodes in Rechargeable Mg Batteries, Chem. Mater. 2004, 16, 2832-2838.
